# DynamicBUS: restoring temporal dynamics from static ultrasound for improved breast cancer diagnosis

**DOI:** 10.1038/s41523-026-01038-4

**Published:** 2026-08-28

**Authors:** Zhikai Yang, Yaofang Liu, Tianhao Bai, Ander Biguri, Haoyuan Chen, Yonghao Li, Carola-Bibiane Schönlieb, Örjan Smedby, Raymond Chan, Rodrigo Moreno

**Affiliations:** 1 Department of Biomedical Engineering and Health Systems, KTH Royal Institute of Technology, Stockholm, Sweden; 2 City University of Hong Kong, Hong Kong, China; 3 COCHE, Hong Kong, China; 4 Department of Ultrasound, Aviation General Hospital, Beijing, China; 5 University of Cambridge, Cambridge, UK; 6 School of Biomedical Engineering & State Key Laboratory of Advanced Medical Materials and Devices, ShanghaiTech University, Shanghai, China; 7 Lingnan University, Hong Kong, China; 8 Department of Neurobiology, Care Sciences and Society, Karolinska Institute, Stockholm, Sweden

**Keywords:** Computational biology and bioinformatics, Engineering, Health care, Mathematics and computing, Medical research

## Abstract

The clinical practice of archiving static 2D images from dynamic breast ultrasound (BUS) examinations loses vital temporal information, limiting computer-aided diagnosis systems. We challenge this constraint by proposing a novel framework that computationally recovers lost temporal dynamics to enhance diagnostic accuracy. Our framework first synthesizes a BUS video from a static key frame using a purpose-built generative model. We introduce a key-frame conditioning strategy to ensure the anatomical fidelity of the lesion is preserved while generating useful dynamic cues. Subsequently, the high-fidelity static image and the synthesized video are fed into Image Video network(IV-Net), a dual-branch fusion network that integrates pristine spatial details with recovered temporal context for robust classification. Evaluated on internal and external datasets, our framework outperforms methods relying solely on static images, achieving AUC of 94.13% and 82.55%, respectively. Furthermore, a reader study indicates the generated videos are indistinguishable to experts and improve diagnostic performance. Overall, our method demonstrates generative model potential to restore lost information, paving the way for reliable BUS diagnostics.

## Introduction

Breast cancer is the most prevalent and threatening disease in females^1^. Ultrasound imaging is one of the most widely used screening techniques for breast cancer. Radiologists use a transducer across the tissue to observe lesion morphology, echogenicity, and deformation from different points of view. Thus, diagnosing with breast ultrasound (BUS) inherently involves dynamic imaging. This spatio-temporal information is used by clinicians to distinguish the possibility of malignant and benign lesions. Unfortunately, in clinical practice, only a few representative static 2D frames are usually archived in a patient’s health records, while the dynamic video is discarded^2^. The Automated Breast Ultrasound (ABUS) can automatically capture full volumetric data blocks^3^, but this technology is still not prevalent in most institutions.

Within the domain of computer-aided breast ultrasound classification, various methodologies have been extensively explored, including radiomics-based approaches^4^ and deep learning-based methods^5–12^. While static BUS image classification has been widely studied, a growing body of evidence highlights the limitations imposed by single-frame analysis. In contrast, methods utilizing BUS videos show improved diagnostic outcomes^13–17^. Videos inherently contain more information, as the integration of spatial and temporal features offers greater insight into lesion characteristics like stiffness or motility patterns. However, the practical utility of these video-based models is severely hampered by the clinical data reality, creating a clear need for methods that can bridge this gap.

Deep learning techniques, such as Convolutional Neural Networks (CNNs), Long Short-Term Memory networks (LSTMs), and self-attention models, have shown remarkable capabilities in extracting complex patterns from video data^18^. These models can automatically learn relevant features from sequences of frames, capturing both spatial and temporal characteristics. Our work builds on this potential but obviates the need for pre-existing video datasets by generating the temporal dimension itself.

In area of video generation models, recent advancements in diffusion models have revolutionized generative modeling, particularly in image synthesis, by employing iterative noise reduction processes to generate high-fidelity samples^19,20^. While extending this framework to video generation has shown promise, existing models often struggle to capture intricate, long-range temporal dynamics^21–29^. For the task of I2V generation, methods like DynamiCrafter^30^ and I2V-Adapter^31^ have made significant progress, yet often face challenges in preserving high fidelity to the conditioning image while generating realistic motion^32,33^.

In the medical imaging domain, generative models have been explored for applications such as endoscopy, cardiac ultrasound, and fundus video generation^34–39^. However, the unique challenge of generating plausible BUS videos from static images for diagnostic purposes remains an unexplored and critical area of research. Our work is the first to address this specific clinical need, adapting the powerful FVDM^40^, whose vectorized timestep mechanism is particularly adept at modeling the complex frame interdependencies and supports full preservation of the conditioning image required for this task. This raises the question of whether these generated breast ultrasound videos can provide additional information for clinical diagnosis.

In this work, we provide a possible answer by proposing a novel, integrated framework that synergistically combines generative video synthesis with a fusion classification network tailored for BUS. The framework consists of two components:

1. *A Temporal Synthesis Module:* We leverage the power of diffusion models to generate a realistic, coherent BUS video from a single static input image. Critically, we introduce a novel key-frame conditioning strategy based on the Frame-Aware Video Diffusion Model (FVDM)^40^. Unlike conventional methods that condition on the first frame, our approach ensures the central, diagnostically critical frame remains true to the source image, while plausible temporal dynamics are synthesized around it. This approach better resembles the exploration strategy used by clinicians.
2. *A Spatio-Temporal Fusion Classifier:* To effectively utilize this combination of real and synthetic data, we designed an Image Video network (IV-Net), a dual-branch network. One branch processes the original, high-fidelity static image to extract precise spatial features, while the other branch analyzes the generated video to capture the synthesized temporal dynamics. To better fuse the image and video information, we design a bidirectional cross-attention (BCA) mechanism. The information from both modalities is then fused to render a final, more informed classification.

This holistic approach transforms a static image problem into a dynamic video analysis problem in the absence of original video data. To our knowledge, this is the first work to explore and validate the concept of generative temporal recovery for BUS lesion classification.

In summary, our main contributions are:

- We propose a new computer-aided diagnostic paradigm that recovers and leverages lost temporal information from static BUS images, comprising a video generation module and a fusion classification network. To the best of our knowledge, this is the first framework to use synthesized video for BUS classification.
- We introduce a key-frame conditioning strategy for diffusion-based image-to-video (I2V) generation.
- We developed an image-video classification network (IV-Net). This dedicated dual-branch network effectively fuses information from a real static image and a generated video, demonstrating a novel approach to integrating generative data into discriminative tasks.

## Experiment and results

Our experiments consisted of two parts. The first part evaluated the quality of the generated BUS video sequences by comparing our proposed video-generation framework with existing state-of-the-art approaches. The second part assessed the classification performance of our method by benchmarking it against established BUS classification methods.

### Experiment setting

The model was implemented in PyTorch and trained on an NVIDIA A100 GPU with 80 GB of memory. We adopted the AdamW optimizer, using a learning rate of 1*e*^−4^ for the diffusion-based generation model and 3*e*^−4^ for the classification model. The generation model was trained from scratch for 100,000 steps with a batch size of 4, while the classification model was trained for 100 epochs with a batch size of 32.

In the video generation stage, the Breast Ultrasound Video (BUSV) dataset^41^ was used. It contains breast lesion ultrasound 188 video clips, including 113 malignant and 75 benign cases. The 148 cases for training and 40 cases for validation for the video generation model. In total, these videos have 25,272 images. Each video provides a complete scan of the tumor, from its appearance to its largest section and subsequent disappearance. In the classification stage, the BUSBRA^42^ and BrEaST^43^ datasets were used. All classification models were conducted five-fold cross-validation in the BUSBRA dataset and externally evaluated on the BrEaST dataset. Table 1 shows the data distribution of these datasets.

**Table 1:** Benign and malignant class distribution in BUSBRA and BrEaST Datasets

Classification category	BUS-BRA	BrEaST
Benign images	857	154
Malignant images	410	98

The video generation model is trained on 16-frame clips of size 256 × 256 with an interval of 3 frame and without lesion class condition, extracted from the BUSV dataset. Given each frame in the dataset, we center-crop and resize it, and then construct 16-frame video clips without class labels. During FVDM training, vectorized timesteps are sampled with PTS to avoid directly optimizing over the full combinatorial timestep space. During DynamicBUS inference, the trained generator is used with the key-frame anchoring schedule described above; no additional key-frame-specific fine-tuning is performed.

By using the trained video generation model, we generated the video from the static BUS dataset. As the BUS contains heterogeneous noise, we applied a low-pass filter and histogram equalization to enhance image quality.

For the video generation experiment, the Fréchet Video Distance (FVD)^44^, Peak Signal-to-Noise Ratio (PSNR), Structural Similarity Index Measure (SSIM), Learned Perceptual Image Patch Similarity (LPIPS)^45^, and Deep Image Structure and Texture Similarity (DISTS)^46^ were used to evaluate the generated video quality.

For the classification experiment, the Precision (PRE), Sensitivity (SEN), the area under the ROC curve (AUC), F1 Score, and Matthews correlation coefficient (MCC) were used for evaluation.

### Video generation performance

To determine the optimal configuration of our I2V generation approach, we conducted an ablation study to compare it with baselines and verify the superiority of our proposed approach, as well as a dataset comparison to assess generalization.

**Fig. 1:**
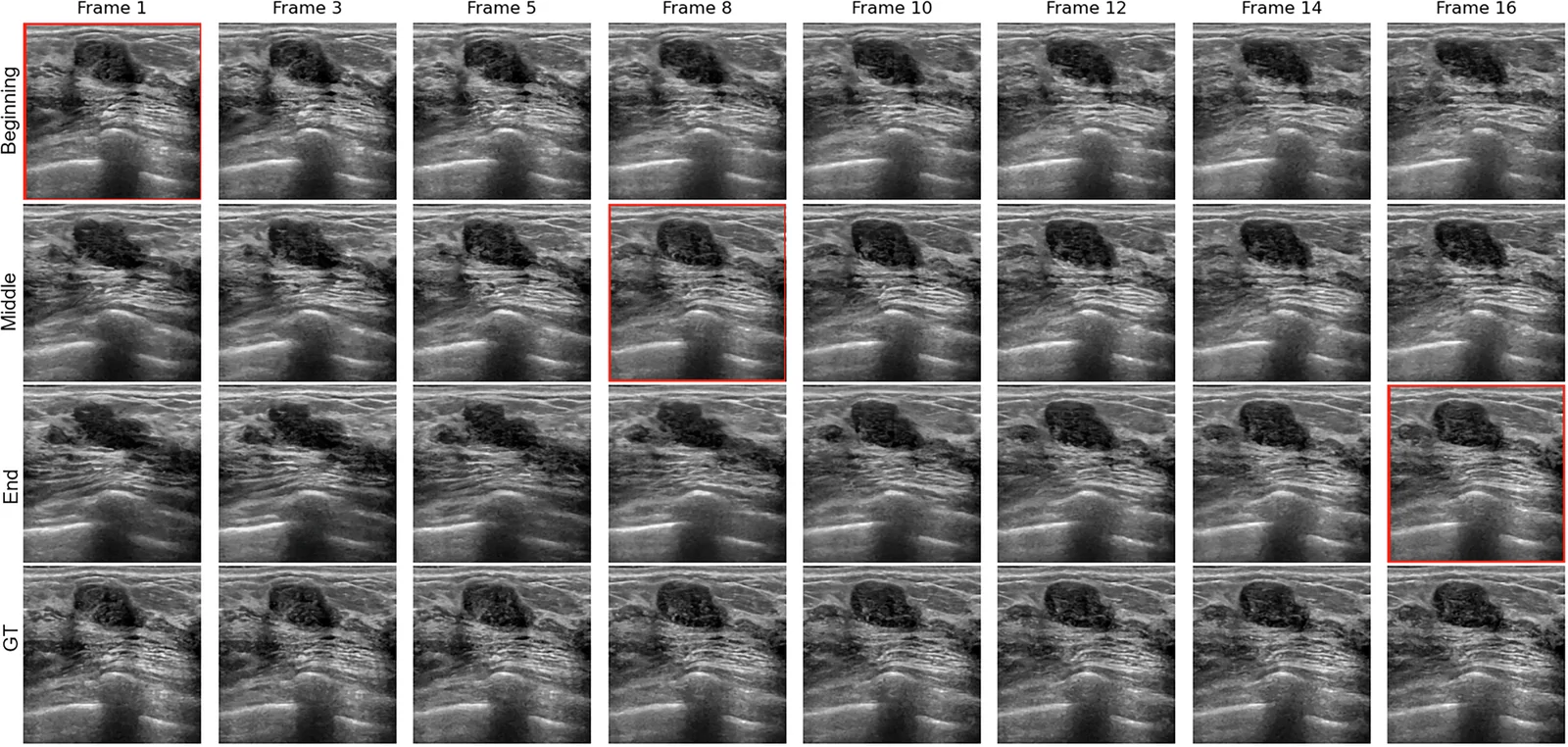
Visualization of the generated video with different condition input and real video. From top to bottom, the generation model takes key images at the beginning, middle, and end positions, which are highlighted with red boxes.

We investigated the effectiveness of our proposed method with ablation studies using the FVD metric (Fig. 1). Figure 1a shows the ablation study for the image conditioned on different frame positions at inference. Specifically, video samples are 16 frames in length. Thus, the conditioning positions range from 1 to 16, representing conditioning from the first frame to the last. The figure shows that our proposed key-anchoring strategy (conditioning on the middle frame) surpasses all other conditioning positions. Fig. 1b compares our I2V generation results with unconditional methods such as original FVDM^40^ and Latte^25^, which directly generate videos from noise. As shown in Fig. 1b, the image condition provides more realistic generation results than generating videos from scratch.

We also compared our proposed method with different I2V generation methods, including both zero-shot and specifically trained methods. As shown in Table 2, the results demonstrate that our method outperforms state-of-the-art video generation methods. Specifically, we trained the MCVD model^47^ on the same dataset from scratch and also list the results for large-scale SOTA models with their pretrained checkpoints. Our method achieved the lowest FVD score of 118.31, indicating higher video quality and better alignment with real video distributions. Additionally, our method attained the highest PSNR (21.01) and SSIM (0.44) scores, signifying superior image fidelity and structural similarity to the ground truth. The model also achieved the lowest LPIPS (0.25) and DISTS (0.16) scores, reflecting enhanced perceptual quality and texture preservation. These results highlight the effectiveness of our proposed approach in bridging the gap between static and dynamic ultrasound imaging.

**Table 2:** Comparisons with other I2V generation methods

Method	FVD (*↓*)	PSNR (*↑*)	SSIM (*↑*)	LPIPS (*↓*)	DISTS (*↓*)
MCVD^47^	639.97	15.79	0.37	0.38	0.24
I2VGen-XL^32^	621.92	16.62	0.32	0.48	0.30
SVD-XT^57^	196.92	19.10	0.38	0.36	0.21
LTX-Video^58^	318.56	18.17	0.42	0.46	0.28
CogVideo^59^	483.86	17.12	0.33	0.45	0.26
Ours	**118.31**	**21.01**	**0.44**	**0.25**	**0.16**

We visualized the generated video sequences for comparison, including videos generated with different frame orders, videos from different static image datasets, and a comparison with the ground truth. The image is surrounded by the red line indicating the proposed method’s reference input.

**Fig. 2:**
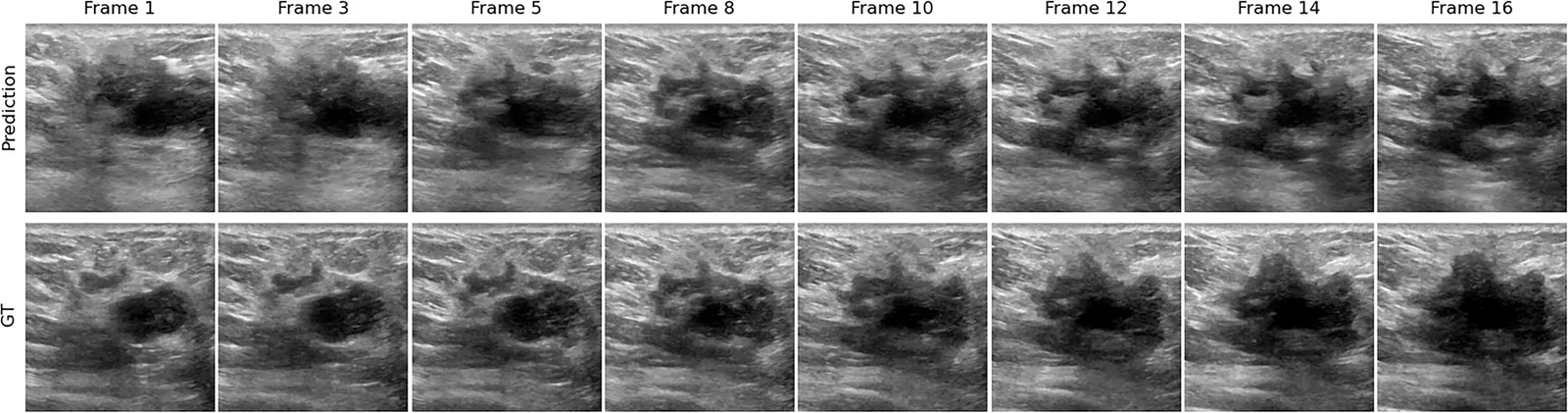
Visualization of our method generated breast ultrasound video sequence compared with the ground truth video sequence.

Figure 2 illustrates the generated video with different frame positions as reference inputs together with the real video sequence. We notice that with different conditioning positions input, result in different generated videos. The proposed key-frame conditioning strategy yields more feasible information that aligns with the original static image, as it can be closer to the original static image from both temporal directions.

**Fig. 3:**
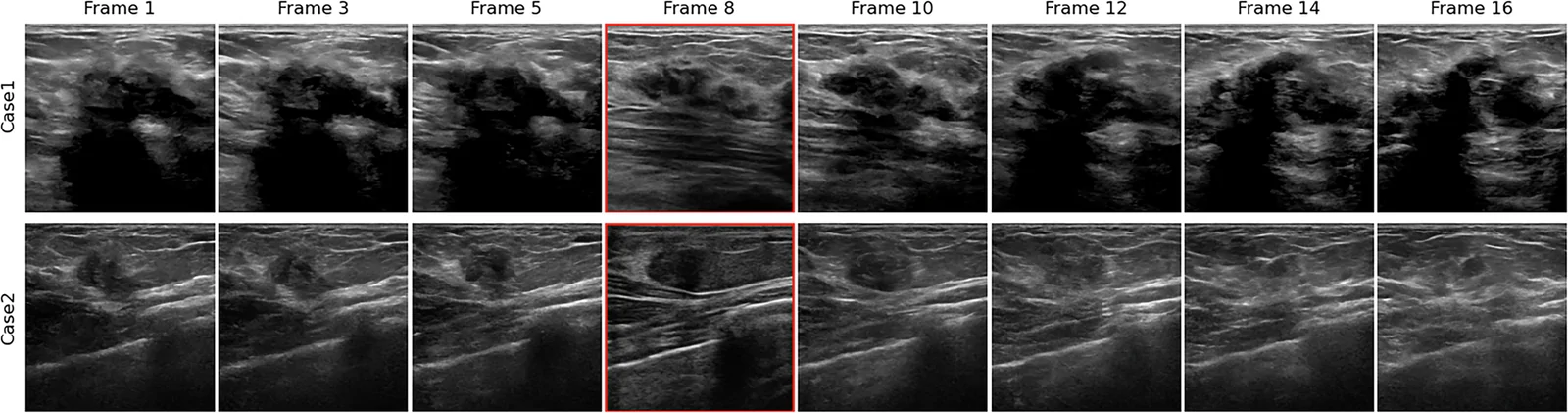
Visualization of videos generated from static images with the proposed method. The red boxes mean conditional input.

Figure 3 shows the generated BUS video compared to the real video. It demonstrates that the generated video of the tumor area is closer to the ground truth video at frames 10 to 16, recovering most of the original information. Frames from 1 to 5 show more variance, but the overall layout still aligns well.

**Fig. 4:**
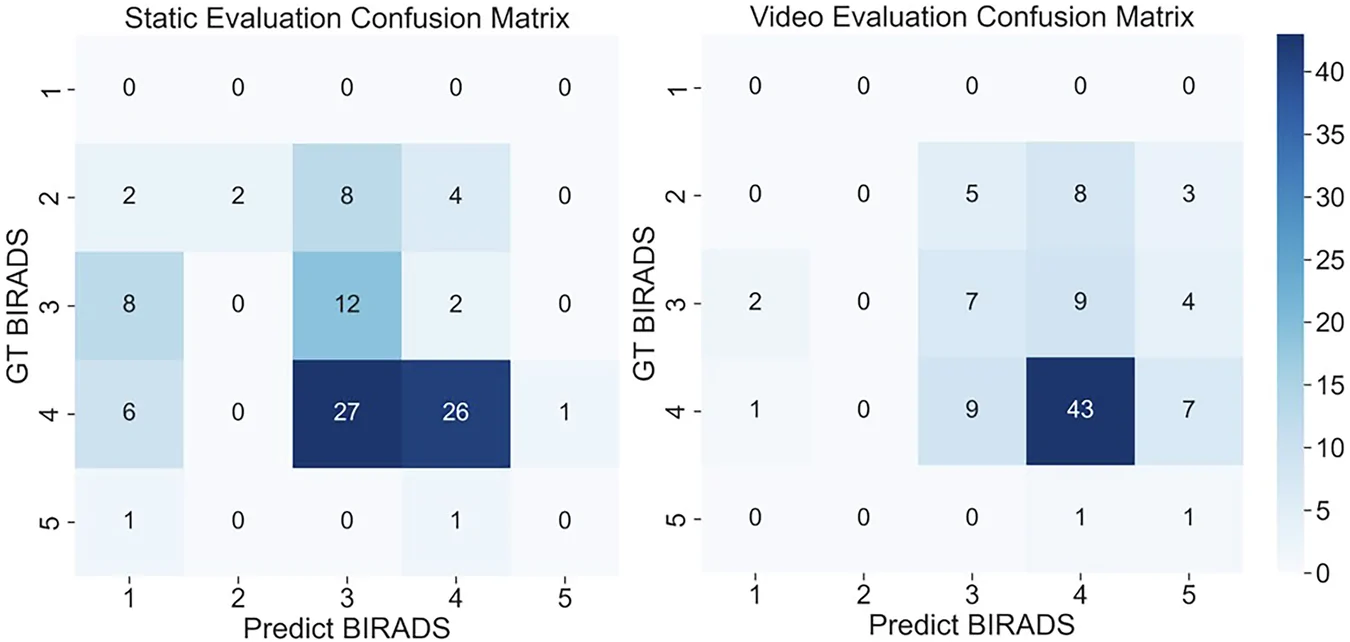
The confusion matrix of the radiologist reader study for predicting BI-RADS scores using static images (left) and adding a generated video (right).

Figure 4 visualizes the generated video sequence from the static image dataset. These cases demonstrate that while the tumor is not clearly visible in the static image, the dynamic image reveals a larger range and shows it invading the surrounding area.

### Reader study

To further validate the diagnostic plausibility of the generated BUS videos, we conducted a blinded reader study with an experienced breast ultrasound radiologist (5 years of experience). We performed two studies: Synthesis Video Quality Evaluation and Diagnosis Evaluation. Following a similar procedure applied in a previous study^48^, we designed an experiment to assess whether an expert could distinguish between real BUS videos and those generated by our method, and to evaluate the perceived clinical realism of the synthetic sequences. We sampled a balanced set of 100 paired BUS videos from the BUSV dataset and 100 synthetic videos generated using our method. The distribution of benign and malignant lesions was matched across the two groups. To avoid bias, no real and generated pair from the same lesion was included in the same reader’s session.

In the evaluation procedure, each video was presented in a randomized order using a designed experiment platform with standard ultrasound playback controls (start/stop, loop, zoom). For each case, readers were asked to label the video as real or synthetic and rate their confidence on a five-level Likert scale. The radiologist achieved an average accuracy of 58.2% in distinguishing real from synthetic videos, only slightly above the chance level (50%), with no significant bias toward either class. Confidence was higher when the reader judged videos as real, but both real and synthetic videos received comparable diagnostic quality scores (median Likert rating = 3). These results suggest that our generated BUS videos are largely indistinguishable from real ones in terms of visual plausibility and clinical fidelity.

A diagnostic evaluation experiment was conducted to quantify the incremental diagnostic benefit of the generated BUS video compared with a single static image. The experiment was as follows: first, the static breast ultrasound image was presented, and the radiologist was asked to assign a Breast Imaging Reporting and Data System (BI-RADS) score. Then, the generated video of 16 frames was played, and the radiologist was allowed to change the original BI-RADS score. The BrEaST^43^ dataset was used for this experiment.

**Fig. 5:**
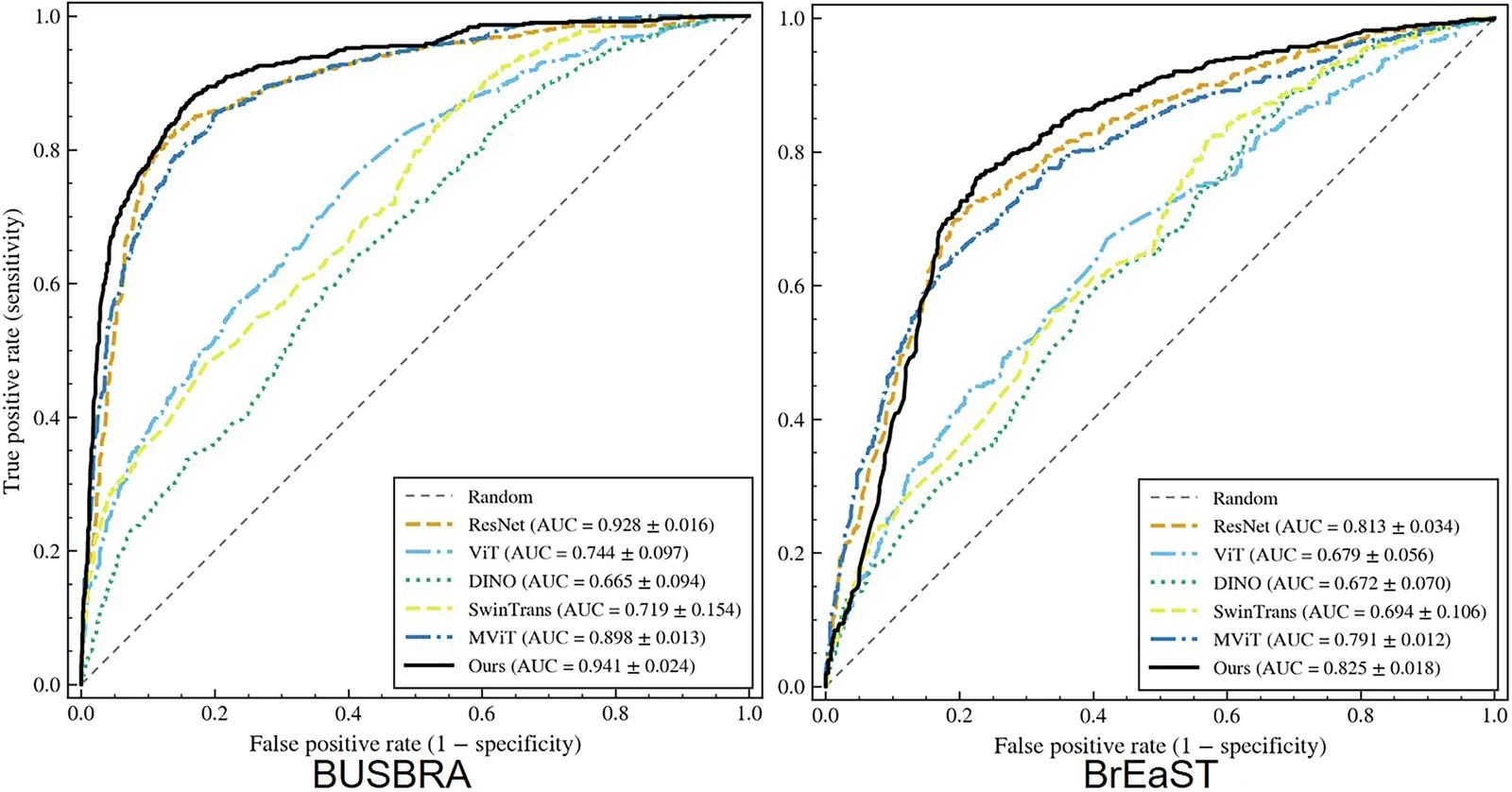
Comparison of the different classification methods ROC curve at BUSBRA and BrEaST dataset.

Figure 5 shows the radiologist’s predicted confusion matrix for static images and breast ultrasound videos. Classes 4A, 4B, and 4C were combined into a single class 4. We found that the overall accuracy improved from 40% with static images to 51% with the image-video pair.

In this experiment, only ultrasound breast images were used, without incorporating clinical information or lesion location data. This limitation resulted in reduced classification performance, as contextual and positional cues are known to contribute to more accurate diagnostic decisions. We acknowledge that the radiologists’ BI-RADS performance in our reader study appears relatively low. This can be attributed to the fact that the BI-RADS annotations in the dataset were assigned during the actual clinical examination, where radiologists had access to additional patient information and real-time scanning feedback. In contrast, our reader study restricted radiologists to static images without this supplementary diagnostic context, inherently affecting their performance.

### Comparison of classification performance with different methods

To assess the effectiveness of the proposed method, we compared the different breast ultrasound classification models, including image-based and video-based classification models with five-fold cross-validation at BUSBRA dataset and externally evaluated on BrEaST dataset. We compared our proposed IV-Net method with image-only: ResNet^49^, Vision Transformer (ViT)^50^, DINOv2^51^, SwinTrans^52^, video-only: MViT^53^ with pretrained weight as shown in Table 3. In our proposed method, we use ResNet and MViT as image and video encoder. This benchmark model was implemented using the PyTorch library. The paired *t*-test was used to between the our method and best performed method at each metric. The results shows the proposed method achieve best performance at most metric both internal and external evaluation.

**Table 3:** Performance comparison of different breast ultrasound classification methods on the BUSBRA

Method	Input	PRE (%)	SEN (%)	AUC (%)	F1 (%)	MCC (*↑*)
*Cross Validation: BUSBRA*						
ResNet	Image	86.27 ± 2.45	85.54 ± 9.81	92.81 ± 1.63	76.93 ± 5.96	65.99 ± 9.07
ViT	Image	50.53 ± 32.69	**91.60 ± 8.49**	74.41 ± 9.74	57.21 ± 7.44	24.84 ± 20.96
DINO	Image	47.80 ± 30.50	90.94 ± 7.56	66.50 ± 9.40	54.10 ± 4.78	18.59 ± 15.83
SwinTrans	Image	50.72 ± 33.57	89.30 ± 9.69	71.90 ± 15.39	59.56 ± 12.52	27.87 ± 28.17
MViT	Video	84.98 ± 1.77	82.70 ± 1.55	89.77 ± 1.31	76.99 ± 2.66	65.21 ± 4.40
**Ours**	**Both**	**88.07 ± 2.85**^a^	85.34 ± 1.17	**94.13 ± 2.42**^a^	**81.59 ± 4.07**^a^	**72.39 ± 6.55**^a^
*External Validation: BrEaST*						
ResNet	Image	75.56 ± 3.39	82.65 ± 9.98	81.30 ± 3.43	68.05 ± 3.57	44.48 ± 6.53
ViT	Image	46.95 ± 26.20	82.24 ± 19.27	67.94 ± 5.58	57.92 ± 4.20	17.96 ± 15.25
DINO	Image	46.79 ± 25.93	83.06 ± 14.15	67.18 ± 7.01	59.17 ± 3.37	18.76 ± 15.90
SwinTrans	Image	48.45 ± 27.40	79.39 ± 18.05	69.44 ± 10.56	60.04 ± 5.12	22.75 ± 19.66
MViT	Video	75.75 ± 0.70	67.55 ± 7.23	79.05 ± 1.20	67.95 ± 1.30	48.47 ± 1.55
**Ours**	**Both**	**79.75 ± 1.62**^a^	**83.67 ± 4.52**	**82.55 ± 1.79**^a^	**74.34 ± 1.86**^a^	**56.09 ± 3.25**^a^

^a^Indicates that our method achieves statistically significant improvement (*p* < 0.05) against the highest-performing baseline using a paired *t*-test.

**Fig. 6:**
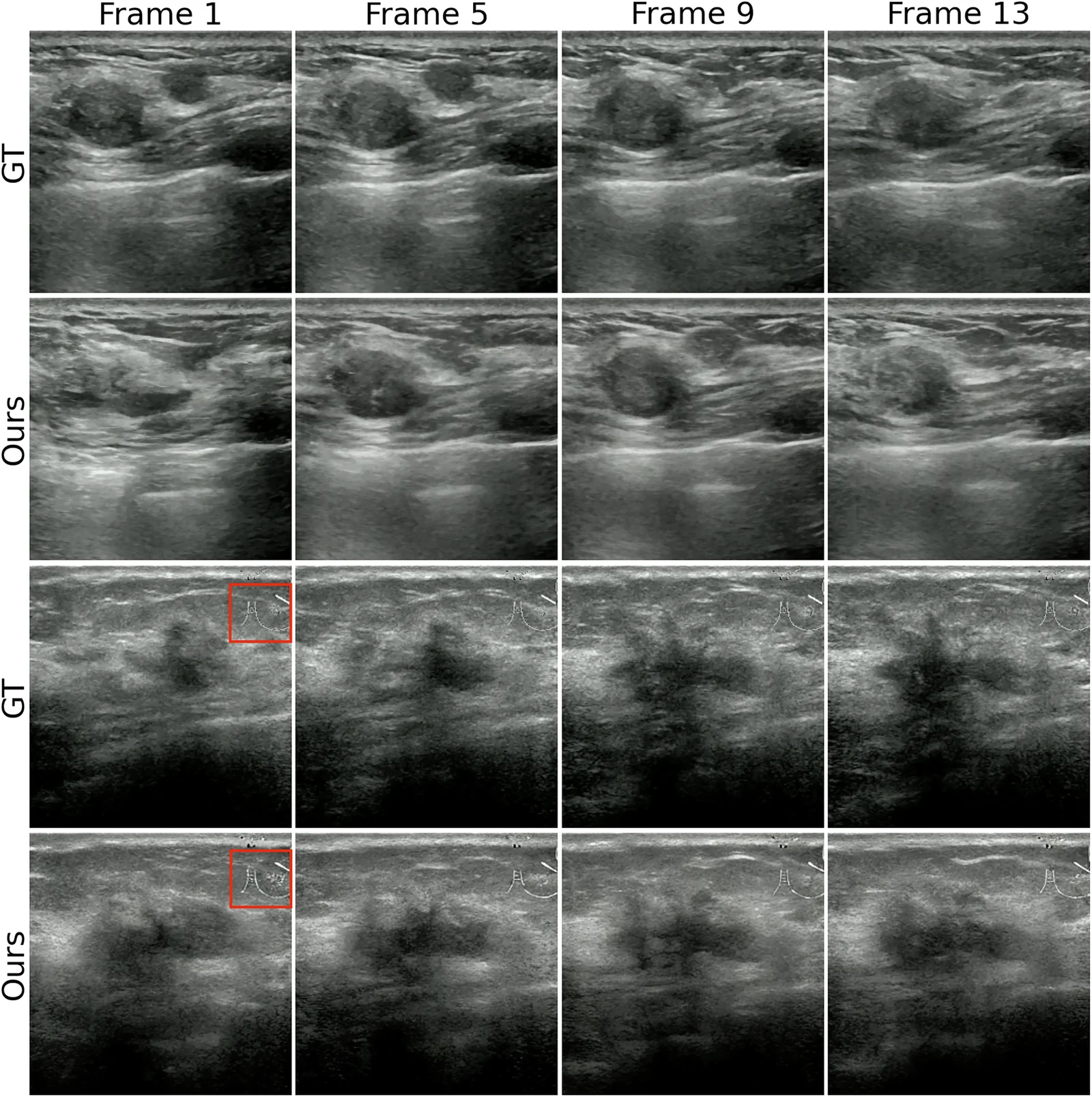
Visualization of limitations in the generated videos. The two rows show a similar comparison for a video with minor color shifts and chart overlays (within the red boxes), where the synthetic video exhibits flickering of chart elements.

Figure 6 shows the different methods’ ROC curve (Receiver Operating Characteristic) for the BUSBRA and BrEaST datasets. Our method significantly outperforms the image and video-based methods on both datasets, achieving an AUC of 94.13% ± 2.42% and 82.55% ± 1.79% (*p* < 0.05). This indicates the added value of considering both the image and the generated video as input.

Additionally, the video-based method performs better than some of the image-based methods, which demonstrating that the generated video sequence is helpful for diagnosis. Furthermore, our proposed IV-Net performs better than only image or video-based methods, which shows that it is important to incorporate the original static image for diagnosis.

### Comparison of classification performance under different settings

The proposed classification model was evaluated with 4, 8, and 16 frames of the generated video to determine the optimal sequence length for breast ultrasound classification. The 4- and 8-frame sequences were obtained by evenly downsampling the original 16-frame videos. The results in Table 4 indicate that while reducing the number of frames introduces minimal performance degradation, using 16 frames achieves the best overall performance. This suggests that our video generation method effectively captures and generates useful temporal information for classification.

**Table 4:** Classification performance with different numbers of frames for breast ultrasound image-video classification at BUSBRA dataset

Frames	PRE (%)	SEN (%)	AUC (%)	F1 (%)	MCC (*↑*)
4	86.23 ± 2.56	73.45 ± 15.32	91.69 ± 3.00	75.41 ± 8.25	66.73 ± 7.33
8	87.85 ± 2.85	85.00 ± 4.75	93.93 ± 1.94	81.04 ± 4.61	71.70 ± 7.11
16	**88.07 ± 2.86**	**85.34 ± 1.17**	**94.13 ± 2.42**	**81.59 ± 4.07**	**72.39 ± 6.55**

We also investigated different fusion strategies for effectively combining image and video features. As shown in Table 5, we compared early fusion, late fusion, and hybrid fusion mechanisms. The results show that late fusion outperforms early fusion on most metrics, while hybrid fusion achieves the overall best performance, highlighting the importance of flexible feature integration in image-video paired classification.

**Table 5:** Classification performance of different fusion strategies for image-video (Early, Late, Hybrid Fusion)

Method	PRE (%)	SEN (%)	AUC (%)	F1 (%)	MCC (*↑*)
Early	87.13 ± 2.24	78.59 ± 4.63	92.95 ± 1.47	79.73 ± 3.53	70.42 ± 5.21
Late	87.51 ± 2.79	79.10 ± 7.42	93.26 ± 2.02	79.96 ± 4.58	71.01 ± 6.61
Hybrid	**88.07 ± 2.85**	**85.34 ± 1.17**	**94.13 ± 2.42**	**81.59 ± 4.07**	**72.39 ± 6.55**

We also compare the performance with different image encoder backbone incorporating the proposed method as shown in Table 6. With the combination of generated video, the proposed method could improve the classification performance compare to the just image classifier and single image input.

**Table 6:** Performance comparison of different breast ultrasound classification methods at the BUSBRA dataset with different image encoder backbone

Method	PRE (%)	SEN (%)	AUC (%)	F1 (%)	MCC (*↑*)
ResNet	**88.07 ± 2.85**	85.34 ± 1.17	**94.13 ± 2.42**	**81.59 ± 4.07**	**72.39 ± 6.55**
ViT	78.40 ± 2.40	85.18 ± 4.83	83.71 ± 3.78	65.74 ± 3.68	46.63 ± 6.26
DINO	50.59 ± 32.87	**85.38 ± 13.35**	72.66 ± 12.51	58.82 ± 8.74	28.13 ± 23.53
SwinTrans	86.40 ± 1.85	84.34 ± 4.61	93.81 ± 1.18	78.61 ± 3.39	67.99 ± 5.47

## Discussion

We proposed a diffusion-based video generation method that leverages prior BUS video information to assist in diagnosis. This approach offers two key advantages for BUS video generation: (1) preservation of the diagnostic frame at the temporal center and (2) bidirectional anatomical consistency. In this way, static BUS images are transformed into coherent video sequences that capture tissue dynamics critical for malignancy assessment while maintaining diagnostic integrity. To the best of our knowledge, this is the first study to explore the use of generated ultrasound videos for diagnostic purposes, providing a new direction for the application of video generation models in computer-aided diagnosis. We experimented with the generated BUS videos using quantitative and qualitative criteria, and the results show that our proposed method achieves the best video quality.

We also note that allowing an arbitrary frame to serve as the conditioning input is not a trivial extension of first-frame video generation. It changes the task from one-sided extrapolation to two-sided synthesis around a fixed clinical anchor, while requiring the anchor frame to remain unchanged. This creates heterogeneous noise states within one video and increases the optimization space substantially, which is why the vectorized timestep design and PTS are necessary. At the architectural level, frame-wise timesteps are needed so the denoiser can jointly reason over a clean key frame and noisy surrounding frames during bidirectional synthesis.

To assess the value of the generated videos from a clinical perspective, we performed a reader study with an ultrasound radiologist to evaluate both the realism of the videos and whether they could provide additional information for improving diagnosis. We found that the radiologist had low inter-observer variability in the realism reader study. For the diagnosis reader study, the radiologist achieved 51% accuracy with the generated video, compared to 40% with a static image. However, this is still relatively lower than the normal diagnostic procedure. This can be attributed to the fact that the dataset only provides B-mode BUS, whereas BI-RADS diagnosis relies on clinical information, more ultrasound modes (Color Doppler and B-mode), and relative positional information.

We compared our method with several breast ultrasound classification models, and the proposed image-video fusion method consistently achieved the best performance at internal and external dataset evaluation. This demonstrates that accurate video generative models can augment data to provide richer diagnostic cues than static images alone. To identify the optimal integration strategy, we evaluated early, late, and hybrid fusion mechanisms. The results show that hybrid fusion performs best overall, particularly when implemented with a trainable fusion module. We argue that in image-video classification, each modality benefits from independently learning specialized, high-level features before combining them.

**Fig. 7:**
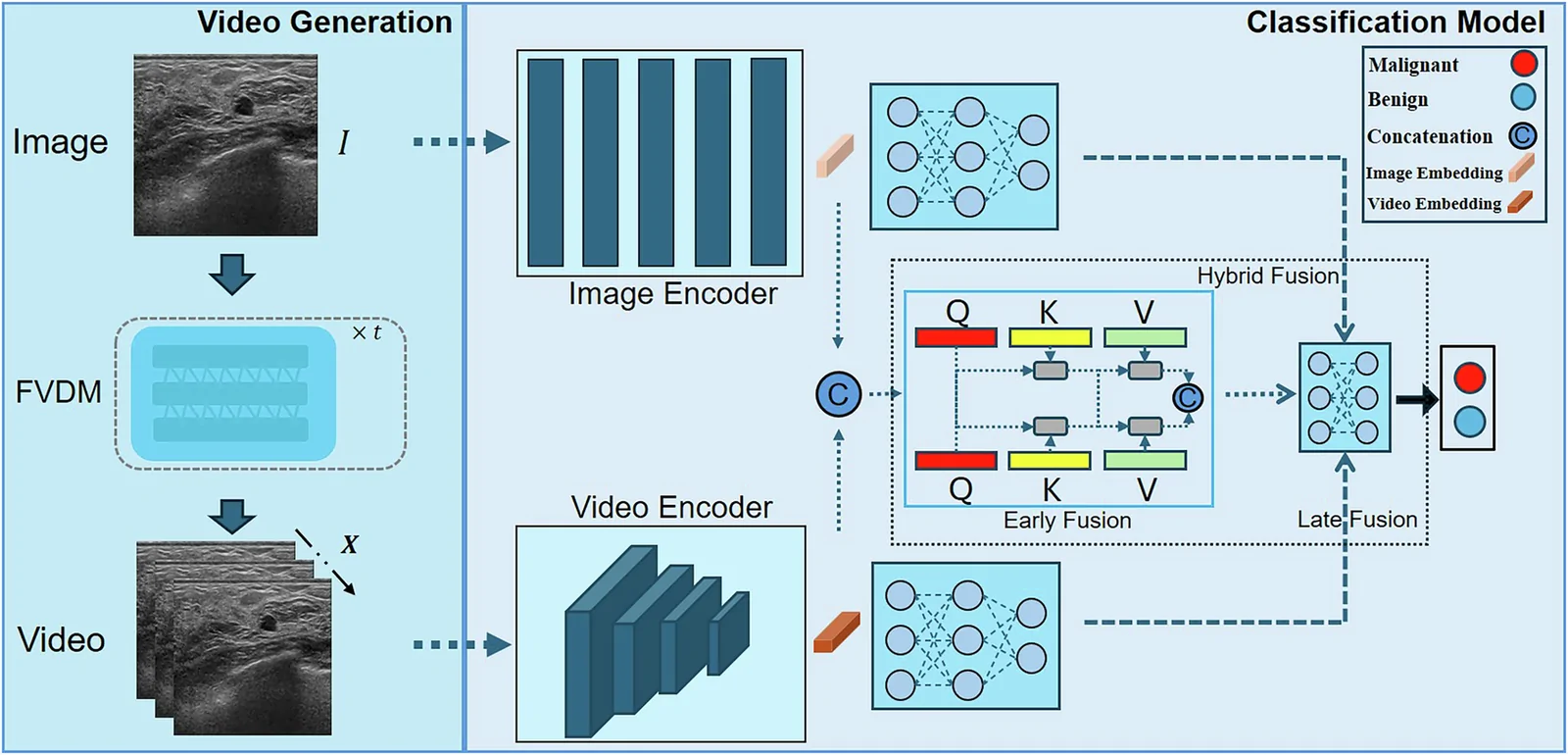
The proposed breast ultrasound classification framework consists of two stages. In the first stage, a diffusion-based model generates a breast ultrasound video from a static image. In the second stage, the generated video and the original static image are used to classify the breast tumor type.

Nevertheless, we acknowledge the following limitations. Firstly, the video generation process is based only on a static image without additional information, which may lead to misleading results. In future work, we plan to incorporate more information to aid BUS video generation, such as lesion area masks, text descriptions, and clinical information. Secondly, video generation models may introduce artifacts or misleading information, which could negatively affect diagnostic decisions. Although the motion and structure of the generated videos are well-aligned with real videos, some noticeable visual artifacts can be observed. In Fig. 7, the synthetic video shows minor color shifts and blurring compared to real ultrasound images. Furthermore, if the original video contains overlays such as measurement lines, these elements can appear to flicker in the generated sequence. These issues are common challenges in generative video synthesis and can likely be mitigated by scaling up training data and computational resources, as well as through further algorithmic refinements. In general, despite these minor imperfections, our method shows potential for improving breast ultrasound diagnosis by recovering lost temporal information. Another issue is that the inference speed of video generation remains a challenge, which is an inherent problem of diffusion models. In future work, we will incorporate anatomical information for video generation and try to accelerate the generation process.

In summary, we addressed the critical issue of information loss in breast ultrasound diagnosis, where dynamic video data is routinely discarded in favor of static images. We introduced a diagnostic framework that computationally recovers these lost temporal dynamics to improve classification accuracy. Our approach first synthesizes a realistic BUS video from a static frame using a diffusion model with a key-frame conditioning strategy to ensure anatomical fidelity. Subsequently, a co-designed dual-branch IV-Net synergistically fuses spatial information from the original image with the recovered temporal context from the generated video. Our experiments show that this integrated approach outperforms methods relying on either static images or pre-existing videos alone.

Future work will focus on extending our framework to generate longer video sequences, accelerating sampling speed and validating its efficacy across a broader range of datasets. Furthermore, this generative restoration paradigm could be extended beyond BUS to other ultrasound applications where temporal data is routinely lost. This research paves the way for a new class of computer-aided diagnosis systems that can overcome fundamental limitations in clinical data archiving.

## Methods

**Fig. 8:**
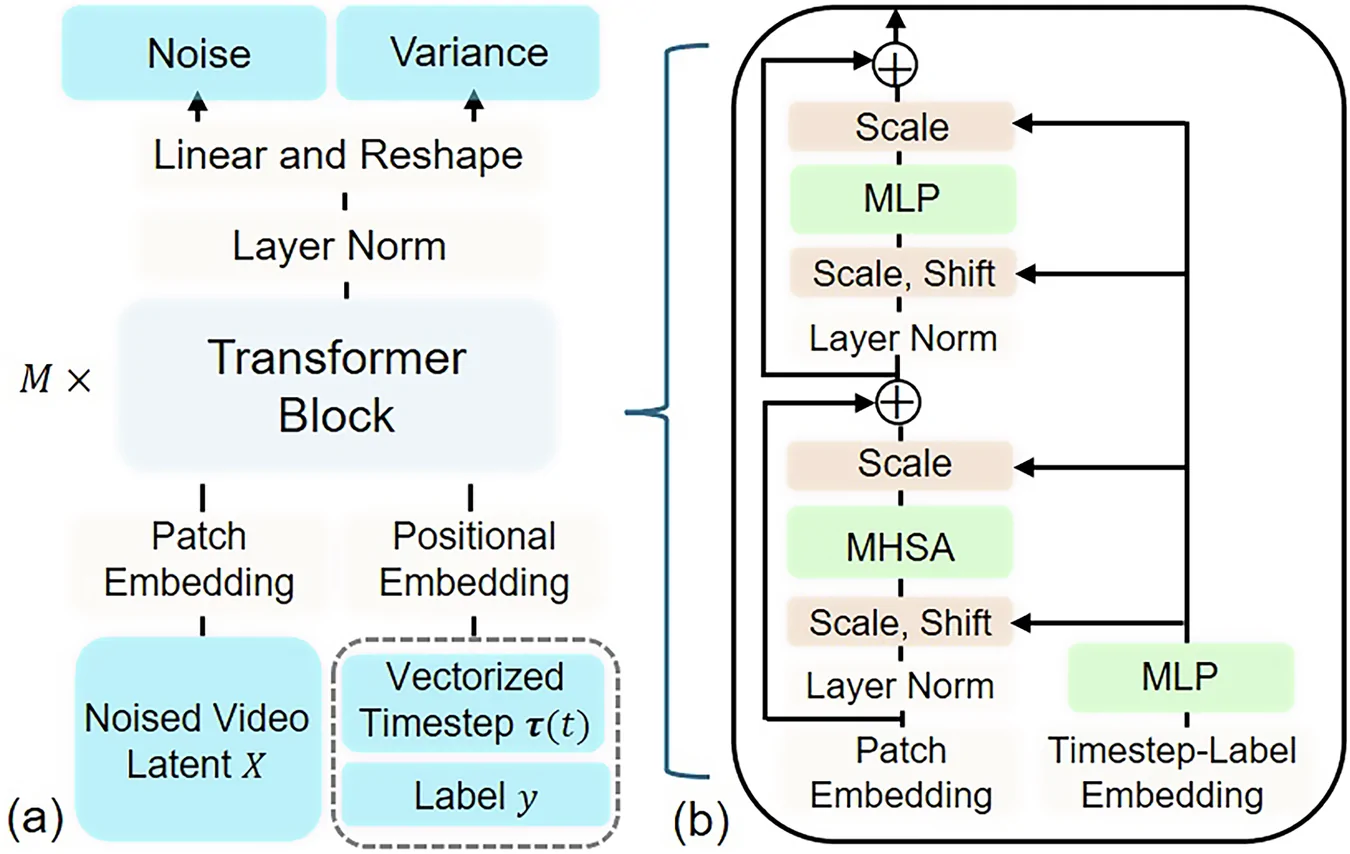
Detailed architecture of the diffusion model. **a** The whole diffusion transformer architecture with components like Patch Embedding, Positional Embedding, Layer Norm, and Transformer Block. **b** Transformer block. MLP and MHSA denote multi-layer perception layer and multi-head self-attention module, respectively.

The proposed framework for BUS classification consists of two main stages: video generation and classification, as shown in Fig. 8. In the first stage, video generation model synthesizes BUS video **X** = [**x**^(1)^, …, **x**^(*N*)^] from the given static image *I*. Then, the generated video, together with the static image, were used as input to classify the breast tumor, described as follows:

1 $${\bf{X}}={\mathcal{G}}(I)$$

2 $$\hat{y}={\mathcal{C}}(I,{\bf{X}})$$

where ${\mathcal{G}}$, $I\in {{\mathbb{R}}}^{w\times h}$, ${\bf{X}}\in {{\mathbb{R}}}^{N\times w\times h}$ and ${\mathcal{C}}$ represent the video generation model, the given image, the generated video and classifier respectively, where *N* represents the total number of frames in the generated video, *w* and *h* denote the height and width. Each image captures a specific lesion or mass region. Given an input static breast ultrasound image *I*, the diffusion model generates a sequence of frames ${\bf{X}}=[{{\bf{x}}}^{(1)},\ldots,{{\bf{x}}}^{(N)}],X\in {{\mathbb{R}}}^{N\times h\times w}$. The $\hat{y}$ is the predicted label (benign or malignant).

In the first stage, we train an FVDM^40^ on BUS video dataset to generate videos. This trained model use a key-frame anchoring schedule at inference to generate a breast ultrasound video from a given static image, such that the image becomes the center frame of the synthesized video sequence. In the second stage, we propose an image-video fusion-based classification model (IV-Net) to predict the tumor type.

### Key frame based breast ultrasound video generation

We present a diffusion based model for synthesizing dynamic BUS videos from static images, addressing the critical need for temporal data in BUS-based cancer classification. The generative model introduces one critical innovation that uses a key frame as a conditional anchor to generate forward and backward frames, diverging from conventional I2V methods, which are conditioned on the first frame and generate forward frames^30,31^. This distinction is clinically important because the archived BUS image is usually the diagnostically representative frame rather than the first frame of the original sweep.

Allowing the conditioning frame to appear at an arbitrary temporal position raises additional design and architectural challenges. At the method level, the model must solve a two-sided temporal synthesis problem: frames before and after the anchor should be mutually consistent while the anchor itself remains anatomically unchanged. At the diffusion level, this requires heterogeneous noise states within the same video, with the key frame kept clean and the remaining frames denoised from noisy states. Conventional video diffusion models (VDM) with a scalar timestep cannot represent this configuration because all frames share the same noise level. At the architecture level, the denoiser should condition cross-frame attention on frame-specific noise levels so that the clean key frame can guide lesion morphology, speckle patterns, and surrounding tissue motion. But the FVDM enables this by replacing the scalar timestep with frame-wise timestep embeddings that modulate the diffusion transformer blocks.

Diffusion models generate data by reversing a continuous stochastic perturbation process^19^. The forward process perturbs data *p*_data_(**x**) to noise via the Stochastic Differential Equation (SDE):

3 $${\rm{d}}{\bf{x}}={\boldsymbol{\mu }}({\bf{x}},t)\,{\rm{d}}t+\sigma (t)\,{\rm{d}}{\bf{w}},$$

In this formulation, the drift coefficient ***μ***(**x**, *t*) governs the deterministic evolution of the data, typically pulling the signal mean towards zero to maintain stability. The diffusion coefficient *σ*(*t*) controls the rate of stochastic perturbation, scaling the random noise injected by the standard Brownian motion **w**. Sampling is performed by reversing this dynamics, governed by the reverse-time SDE involving the score function ${\nabla }_{{\bf{x}}}\log {p}_{t}({\bf{x}})$:

4 $${\rm{d}}{\bf{x}}=[{\boldsymbol{\mu }}({\bf{x}},t)-\sigma {(t)}^{2}{\nabla }_{{\bf{x}}}\log {p}_{t}({\bf{x}})]{\rm{d}}t+\sigma (t){\rm{d}}\bar{{\bf{w}}}.$$

For the standard video diffusion models, it usually applies a scalar timestep *t* uniformly across all frames^27^, restricting temporal flexibility. To capture complex temporal dynamics, FVDM^40^ introduces a vectorized timestep mechanism ${\boldsymbol{\tau }}(t)={[{\tau }^{(1)}(t),\ldots,{\tau }^{(N)}(t)]}^{\top }\in {[0,T]}^{N}$, where each *τ*^(*i*)^ governs the noise level for the *i*-th frame independently. This formulation also makes the training space much longer than that of scalar-timestep VDMs. For example, a 16-frame clip with 1000 diffusion timesteps has 1000^16^ = 10^48^ possible vectorized timestep states, whereas a scalar-timestep VDM has only 1000 timestep states. Directly sampling an independent timestep for every frame can therefore expose the model to an excessively large set of denoising configurations. We therefore follow FVDM and use a probabilistic timestep sampling strategy (PTS). In particular, PTS introduces a probability *p* to govern timestep sampling during training. With probability *p*, different timesteps are sampled for different frames, allowing the model to learn frame-wise independent evolution; while, with probability 1 − *p*, one timestep is sampled and shared by all frames, preserving the standard video denoising setting. Following FVDM, we set *p* = 0.2 in all experiments. This hybrid sampling strategy reduces the burden of the full vectorized timestep space while retaining the temporal flexibility needed for key-frame anchoring and bidirectional generation. Notice that the noise levels per frame are independent, but the generated frames are consistent because the training is performed on consistent video datasets. Consequently, the diffusion dynamics for the *i*-th frame **x**^(*i*)^ follow the frame-specific SDE:

5 $$d{{\bf{x}}}^{(i)}={\boldsymbol{\mu }}({{\bf{x}}}^{(i)},{\tau }^{(i)})dt+\sigma ({\tau }^{(i)})d{{\bf{w}}}^{(i)}.$$

By aggregating frames into a video matrix ${\bf{X}}={[{{\bf{x}}}^{(1)},\ldots,{{\bf{x}}}^{(N)}]}^{\top }\in {{\mathbb{R}}}^{N\times d}$, we can formulate the integrated forward SDE for the entire video sequence:

6 $$d{\bf{X}}={\boldsymbol{U}}({\bf{X}},{\boldsymbol{\tau }}(t))dt+{\mathbf{\Sigma }}({\boldsymbol{\tau }}(t))d{\bf{W}},$$

where ***U*** represents the concatenated drift coefficients, **Σ** is a diagonal matrix of diffusion coefficients determined by ***τ***, and **W** denotes the multidimensional Brownian motion.

As mentioned, I2V methods typically generate forward frames from a conditioning image. Instead, we use a key frame **x**^(*m*)^ at an arbitrary position *m* as the stable anatomical reference. In this study, we set *m* = ⌊*N*/2⌋ because the archived BUS image is typically the representative lesion frame and center anchoring provides a balanced synthesis of preceding and following frames. Specifically, given a static BUS image *I*, we initialize:

7 $${{\bf{x}}}^{(m)}=I\,{\rm{with}}\,{\tau }^{(m)}(t)\equiv 0,$$

while applying standard diffusion to other frames:

8 $${\tau }^{(i)}(t)=t,\quad \forall i\,\ne\,m.$$

This schedule enables bidirectional synthesis video from the anchor image while preserving diagnostic fidelity. Importantly, key-frame anchoring is an inference-time schedule built on the trained FVDM denoiser; we do not add a separate key-frame encoder, conditioning branch, or task-specific fine-tuning stage. By setting the anchor frame’s timestep to zero (*τ*^(*m*)^(*t*) ≡ 0) and applying regular diffusion to other frames, the model treats the input image as a noise-free frame inside the video tensor. The diffusion transformer then uses its native spatio-temporal self-attention to propagate information from this clean anchor to the surrounding noisy frames.

**Fig. 9:**
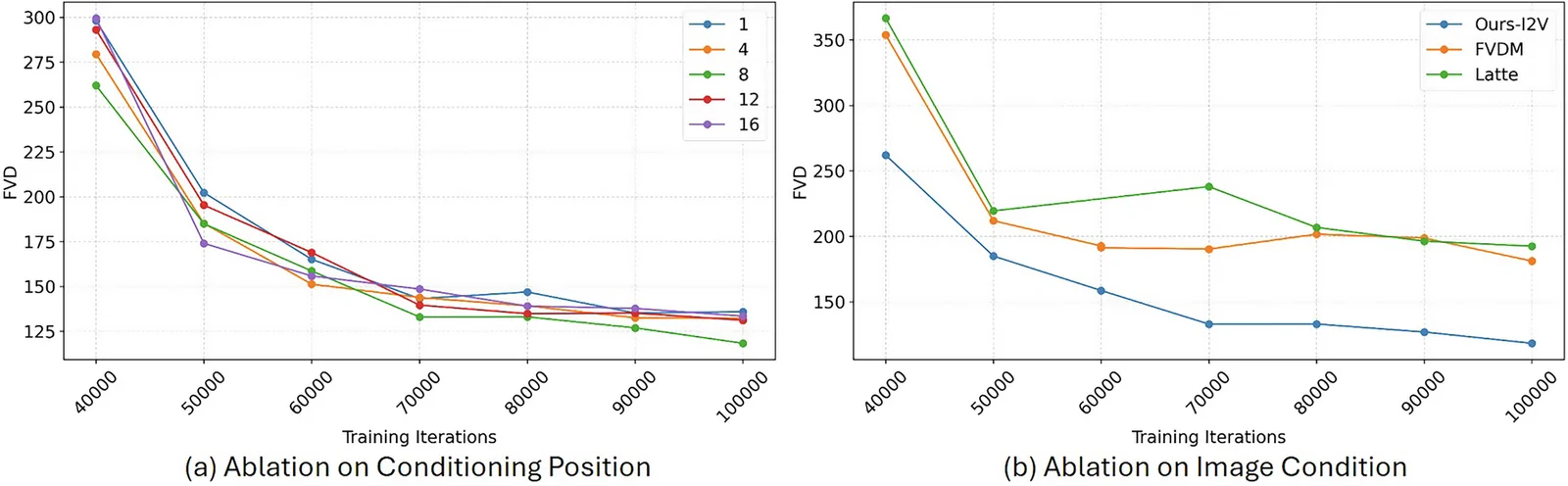
**a** Evolution of FVD during training of FVDM w.r.t. different positions for image condition. The green line (conditioning on the 8th frame) achieves the best performance. **b** Evolution of FVD during training of our I2V method (key-anchoring strategy), FVDM^40^, and Latte^25^.

Figure 9 illustrates the full diffusion transformer architecture based on Ma et al.^25^. The difference is the replacement of the scalar timestep input with a vectorized version. Sinusoidal positional encoding transforms these timesteps to frame-wise embeddings (*N*, *D*), where *D* is the embedding dimension. These embeddings condition the transformer attention and MLP layers via adaLN-Zero^54^, enabling the denoiser to process frames with different noise levels in the same sequence and to use the clean anchor frame for cross-frame noise prediction. By implementing this enhanced method, we convert static breast ultrasound images into dynamic, multi-frame video sequences.

### Image-video classification network

To better process key frame and generated video information, we propose IV-Net shown in Fig. 8 classification model part. The pair key frame image and generated video together are used as input. The IV-Net consists of three components: an image classification branch, a video classification branch, and a feature fusion module. IV-Net is inspired by the two-branch architecture slow-fast video classification method^55^, a model with two streams designed for video understanding tasks. Unlike the slow-fast branches, which capture both slow and fast video sequences, IV-Net’s two branches are used to capture the key breast ultrasound image and generate video information. In the image stream, we used ResNet as the backbone to extract the image feature map ${f}_{I}\in {{\mathbb{R}}}^{c}$ where *c* represents the channel dimension. In the video stream, the MViT^53^ network model is used for extracting spatial and temporal features ${f}_{X}\in {{\mathbb{R}}}^{c}$. After extracting the image and video features, a feature representation fusion module is used to fuse the features.

To better incorporate the extracted features, we propose to use the hybrid fusion module to handle the image-video paired data. It combines the early and late fusion module.

#### Early fusion

In the early fusion strategy, feature-level integration is performed immediately after extracting representations from the image and video branches. We then apply the BCA module to explicitly model interactions between modalities. Fig. 8 shows the early fusion module, which works as follows. First, the features of both branches are projected into a shared latent space $f\in {{\mathbb{R}}}^{c}$ through modality-specific linear layers :

$${q}_{I}={W}_{I}^{Q}{f}_{I},\quad {k}_{I}={W}_{I}^{K}{f}_{I},\quad {v}_{I}={W}_{I}^{V}{f}_{I},$$

$${q}_{X}={W}_{X}^{Q}\,{f}_{X},\quad {k}_{X}={W}_{X}^{K}\,{f}_{X},\quad {v}_{X}={W}_{X}^{V}\,{f}_{X}.$$

where produces query ${W}^{Q}\in {{\mathbb{R}}}^{c\times h}$, key ${W}^{K}\in {{\mathbb{R}}}^{c\times h}$, and value ${W}^{V}\in {{\mathbb{R}}}^{c\times h}$ representations. The image query attends to the video key to obtain video-guided image features, while the video query attends to the image key to obtain image-guided video features. Then, the attended features are computed:

$${\tilde{f}}_{I}={\alpha }_{I}{v}_{X},\quad {\tilde{f}}_{X}={\alpha }_{X}{v}_{I},$$

with

$${\alpha }_{I}={\rm{Softmax}}({q}_{I}{k}_{X}^{\top }),\quad {\alpha }_{X}={\rm{Softmax}}({q}_{X}{k}_{I}^{\top }).$$

Finally, the attended features are concatenated to form the fused representation:

$${f}_{{\rm{early}}}=({\tilde{f}}_{I}\parallel {\tilde{f}}_{X})\in {{\mathbb{R}}}^{2c},$$

where ∥ is a concatenation operation. These features are aggregated and passed through an MLP for classification. By employing bidirectional cross-attention, the model is able to emphasize salient cross-modal interactions and capture complementary dependencies beyond simple concatenation. This attention-based formulation extends the multi-head self-attention mechanism^56^ to explicitly model cross-modal interactions at the feature level, allowing the model to emphasize salient information across modalities. This mechanism enables the model to learn complementary information by allowing each modality to selectively attend to the most relevant features from the other, thereby capturing richer cross-modal interactions than simple concatenation or independent processing.

#### Late fusion

In contrast, the late fusion strategy defers the integration of information until the decision stage. The image and video features *f*_*I*_ and *f*_*X*_ are individually processed by their respective fully connected layers to obtain independent classification logits:

$${\hat{y}}_{I}={{\rm{MLP}}}_{I}({f}_{I}),\quad {\hat{y}}_{X}={{\rm{MLP}}}_{X}({f}_{X}).$$

These logits are then concatenated and fed into a fusion MLP, which acts as a decision-level aggregator:

$$\hat{y}={{\rm{MLP}}}_{{\rm{fusion}}}({\hat{y}}_{I}\,\parallel \,{\hat{y}}_{X}),$$

The fusion MLP consists of three fully connected layers with ReLU activations and dropout for regularization. This structure enables the model to learn a nonlinear mapping from modality-specific decisions to a unified classification output.

The late fusion independent classification logits for image and video features are obtained from their respective fully connected layers. A 3-layer MLP is used to fuse the information at the decision stage, aiming at making decisions based on the results of the two modality branches.

#### Hybrid fusion

Hybrid fusion integrates both strategies by combining interactions at the feature and decision levels. First, an early fused representation is obtained via BCA:

$${f}_{{\rm{early}}}={\rm{BCA}}({f}_{I},{f}_{X}),$$

This representation is aggregated and projected into a compact vector *f*_early_. In parallel, modality-specific features are passed into their respective MLP classifiers to produce logits ${\hat{y}}_{I}$ and ${\hat{y}}_{X}$. The final hybrid representation is then formed by concatenating the early fused feature and the modality-specific logits:

$${f}_{{\rm{hybrid}}}=({f}_{{\rm{early}}}\parallel {\hat{y}}_{I}\parallel {\hat{y}}_{X}),$$

which is passed through a fusion MLP to obtain the final output:

$$\hat{y}={{\rm{MLP}}}_{{\rm{fusion}}}({f}_{{\rm{hybrid}}}).$$

By combining both low-level interactions (early fusion) and high-level decisions (late fusion), this design is expected to provide a more expressive and robust integration mechanism that mitigates the limitations of using either strategy alone.

The binary cross-entropy is used as a loss function: ${{\mathcal{L}}}_{{\rm{BCE}}}=-\left[y\log ({y}^{p})+(1-y)\log (1-{y}^{p})\right]$, where *y*^*p*^ denote the prediction probability.

## Supplementary information


Supplementary Information
Supplementary Information


## Data Availability

This study used datasets from Lin, Zhi, et al. "A new dataset and a baseline model for breast lesion detection in ultrasound videos." International Conference on Medical Image Computing and Computer-Assisted Intervention. Cham: Springer Nature Switzerland, 2022. (https://github.com/jhl-Det/CVA-Net), BUS-BRA: A Breast Ultrasound Dataset for Assessing Computer-aided Diagnosis Systems (https://zenodo.org/records/8231412)^43^.

## Code availability

The code and generated example data are public at https://github.com/minnelab/DynamicBUS.
