# Supplementary material for: DynamicBUS: restoring temporal dynamics from static ultrasound for improved breast cancer diagnosis
Source: NPJ Breast Cancer. 2026 Aug 28;12:112. doi: 10.1038/s41523-026-01038-4 (PMC13522559; doi:10.1038/s41523-026-01038-4)
Supplement: Supplementary file 1 [file 41523_2026_1038_MOESM1_ESM.pdf]

Supplementary Information

Qualitative comparison of breast ultrasound video generation methods

1. Overview

This Supplementary Information provides legends for the Supplementary Movie files accompanying the manuscript. The movies present qualitative results of breast ultrasound image-to-video generation, comparing ground-truth ultrasound sequences with videos synthesized by the proposed method and several baseline methods.

All Supplementary Movie files are provided in MP4 format, which is an accepted journal format. Files are clearly labelled using the naming convention Supplementary_Movie_SXX_<Method>_<Class>_caseN.mp4.

2. Organisation of Supplementary Movies

A total of 72 Supplementary Movies (S01-S72) are included. Movies are grouped by method. For each method (except Latte), four benign cases and four malignant cases are provided. The same case identifiers are used across methods to enable direct side-by-side comparison with the ground-truth videos and with the proposed method.

Class labels: benign = clinically benign cases; malignant = clinically malignant cases. In the original folder layout, these correspond to subfolders "0" (benign) and "1" (malignant).

Note on Latte: Latte samples use an independent naming scheme and therefore do not share the same case identifiers as the other methods; they are provided as representative benign and malignant generated examples.

3. Methods included

Ground Truth (Supplementary Movies S01-S08). Real breast ultrasound video sequences used as reference.

Ours / proposed method (Supplementary Movies S09-S16). Video sequences generated by the proposed method.

CogVideo (Supplementary Movies S17-S24). Videos generated by CogVideo.

FVDM (Supplementary Movies S25-S32). Videos generated by FVDM.

I2VGen (Supplementary Movies S33-S40). Videos generated by I2VGen.

LTX (Supplementary Movies S41-S48). Videos generated by LTX.

MCVD (Supplementary Movies S49-S56). Videos generated by MCVD.

SVD-XT (Supplementary Movies S57-S64). Videos generated by Stable Video Diffusion XT (SVD-XT).

Latte (Supplementary Movies S65-S72). Videos generated by Latte (independent sample naming).

4. Case identifiers used for cross-method comparison

Benign cases (cases 1-4): 263b86b85a58f270; 2cda21c3aab26332; 32a6532065f51cec; 391ae00452fe2e99.

Malignant cases (cases 1-4): 1dc9ca2f1748c2ec; 2a2716116cf2a1da; 2aef523b046e4671; 2c12ff6464cfb1ff.

5. Legends for Supplementary Movies

Each entry below gives the Supplementary Movie ID, file name, method, diagnostic class, and case information.

Supplementary Movies - Ground Truth (real ultrasound videos)

Supplementary Movie S01. File: Supplementary_Movie_S01_GroundTruth_benign_case1.mp4. Method: Ground Truth (real ultrasound videos). Class: Benign. Benign case 1 (case ID: 263b86b85a58f270). Format: MP4. This movie shows a breast ultrasound video sequence from the real clinical acquisition (ground truth).

Supplementary Movie S02. File: Supplementary_Movie_S02_GroundTruth_benign_case2.mp4. Method: Ground Truth (real ultrasound videos). Class: Benign. Benign case 2 (case ID: 2cda21c3aab26332). Format: MP4. This movie shows a breast ultrasound video sequence from the real clinical acquisition (ground truth).

Supplementary Movie S03. File: Supplementary_Movie_S03_GroundTruth_benign_case3.mp4. Method: Ground Truth (real ultrasound videos). Class: Benign. Benign case 3 (case ID: 32a6532065f51cec). Format: MP4. This movie shows a breast ultrasound video sequence from the real clinical acquisition (ground truth).

Supplementary Movie S04. File: Supplementary_Movie_S04_GroundTruth_benign_case4.mp4. Method: Ground Truth (real ultrasound videos). Class: Benign. Benign case 4 (case ID: 391ae00452fe2e99). Format: MP4. This movie shows a breast ultrasound video sequence from the real clinical acquisition (ground truth).

Supplementary Movie S05. File: Supplementary_Movie_S05_GroundTruth_malignant_case1.mp4. Method: Ground Truth (real ultrasound videos). Class: Malignant. Malignant case 1 (case ID: 1dc9ca2f1748c2ec). Format: MP4. This movie shows a breast ultrasound video sequence from the real clinical acquisition (ground truth).

Supplementary Movie S06. File: Supplementary_Movie_S06_GroundTruth_malignant_case2.mp4. Method: Ground Truth (real ultrasound videos). Class: Malignant. Malignant case 2 (case ID:

2a2716116cf2a1da). Format: MP4. This movie shows a breast ultrasound video sequence from the real clinical acquisition (ground truth).

Supplementary Movie S07. File: Supplementary_Movie_S07_GroundTruth_malignant_case3.mp4. Method: Ground Truth (real ultrasound videos). Class: Malignant. Malignant case 3 (case ID: 2aef523b046e4671). Format: MP4. This movie shows a breast ultrasound video sequence from the real clinical acquisition (ground truth).

Supplementary Movie S08. File: Supplementary_Movie_S08_GroundTruth_malignant_case4.mp4. Method: Ground Truth (real ultrasound videos). Class: Malignant. Malignant case 4 (case ID: 2c12ff6464cfb1ff). Format: MP4. This movie shows a breast ultrasound video sequence from the real clinical acquisition (ground truth).

Supplementary Movies - Our proposed method

Supplementary Movie S09. File: Supplementary_Movie_S09_Ours_benign_case1.mp4. Method: Our proposed method. Class: Benign. Benign case 1 (case ID: 263b86b85a58f270). Format: MP4. This movie shows a breast ultrasound video sequence generated by the indicated method for qualitative comparison with the corresponding ground-truth sequence where available.

Supplementary Movie S10. File: Supplementary_Movie_S10_Ours_benign_case2.mp4. Method: Our proposed method. Class: Benign. Benign case 2 (case ID: 2cda21c3aab26332). Format: MP4. This movie shows a breast ultrasound video sequence generated by the indicated method for qualitative comparison with the corresponding ground-truth sequence where available.

Supplementary Movie S11. File: Supplementary_Movie_S11_Ours_benign_case3.mp4. Method: Our proposed method. Class: Benign. Benign case 3 (case ID: 32a6532065f51cec). Format: MP4. This movie shows a breast ultrasound video sequence generated by the indicated method for qualitative comparison with the corresponding ground-truth sequence where available.

Supplementary Movie S12. File: Supplementary_Movie_S12_Ours_benign_case4.mp4. Method: Our proposed method. Class: Benign. Benign case 4 (case ID: 391ae00452fe2e99). Format: MP4. This movie shows a breast ultrasound video sequence generated by the indicated method for qualitative comparison with the corresponding ground-truth sequence where available.

Supplementary Movie S13. File: Supplementary_Movie_S13_Ours_malignant_case1.mp4. Method: Our proposed method. Class: Malignant. Malignant case 1 (case ID: 1dc9ca2f1748c2ec). Format: MP4. This movie shows a breast ultrasound video sequence generated by the indicated method for qualitative comparison with the corresponding ground-truth sequence where available.

Supplementary Movie S14. File: Supplementary_Movie_S14_Ours_malignant_case2.mp4. Method: Our proposed method. Class: Malignant. Malignant case 2 (case ID: 2a2716116cf2a1da). Format: MP4. This movie shows a breast ultrasound video sequence generated by the indicated method for qualitative

comparison with the corresponding ground-truth sequence where available.

Supplementary Movie S15. File: Supplementary_Movie_S15_Ours_malignant_case3.mp4. Method: Our proposed method. Class: Malignant. Malignant case 3 (case ID: 2aef523b046e4671). Format: MP4. This movie shows a breast ultrasound video sequence generated by the indicated method for qualitative comparison with the corresponding ground-truth sequence where available.

Supplementary Movie S16. File: Supplementary_Movie_S16_Ours_malignant_case4.mp4. Method: Our proposed method. Class: Malignant. Malignant case 4 (case ID: 2c12ff6464cfb1ff). Format: MP4. This movie shows a breast ultrasound video sequence generated by the indicated method for qualitative comparison with the corresponding ground-truth sequence where available.

Supplementary Movies - CogVideo

Supplementary Movie S17. File: Supplementary_Movie_S17_CogVideo_benign_case1.mp4. Method: CogVideo. Class: Benign. Benign case 1 (case ID: 263b86b85a58f270). Format: MP4. This movie shows a breast ultrasound video sequence generated by the indicated method for qualitative comparison with the corresponding ground-truth sequence where available.

Supplementary Movie S18. File: Supplementary_Movie_S18_CogVideo_benign_case2.mp4. Method: CogVideo. Class: Benign. Benign case 2 (case ID: 2cda21c3aab26332). Format: MP4. This movie shows a breast ultrasound video sequence generated by the indicated method for qualitative comparison with the corresponding ground-truth sequence where available.

Supplementary Movie S19. File: Supplementary_Movie_S19_CogVideo_benign_case3.mp4. Method: CogVideo. Class: Benign. Benign case 3 (case ID: 32a6532065f51cec). Format: MP4. This movie shows a breast ultrasound video sequence generated by the indicated method for qualitative comparison with the corresponding ground-truth sequence where available.

Supplementary Movie S20. File: Supplementary_Movie_S20_CogVideo_benign_case4.mp4. Method: CogVideo. Class: Benign. Benign case 4 (case ID: 391ae00452fe2e99). Format: MP4. This movie shows a breast ultrasound video sequence generated by the indicated method for qualitative comparison with the corresponding ground-truth sequence where available.

Supplementary Movie S21. File: Supplementary_Movie_S21_CogVideo_malignant_case1.mp4. Method: CogVideo. Class: Malignant. Malignant case 1 (case ID: 1dc9ca2f1748c2ec). Format: MP4. This movie shows a breast ultrasound video sequence generated by the indicated method for qualitative comparison with the corresponding ground-truth sequence where available.

Supplementary Movie S22. File: Supplementary_Movie_S22_CogVideo_malignant_case2.mp4. Method: CogVideo. Class: Malignant. Malignant case 2 (case ID: 2a2716116cf2a1da). Format: MP4. This movie shows a breast ultrasound video sequence generated by the indicated method for qualitative comparison with the corresponding ground-truth sequence where available.

Supplementary Movie S23. File: Supplementary_Movie_S23_CogVideo_malignant_case3.mp4. Method: CogVideo. Class: Malignant. Malignant case 3 (case ID: 2aef523b046e4671). Format: MP4. This movie shows a breast ultrasound video sequence generated by the indicated method for qualitative comparison with the corresponding ground-truth sequence where available.

Supplementary Movie S24. File: Supplementary_Movie_S24_CogVideo_malignant_case4.mp4. Method: CogVideo. Class: Malignant. Malignant case 4 (case ID: 2c12ff6464cfb1ff). Format: MP4. This movie shows a breast ultrasound video sequence generated by the indicated method for qualitative comparison with the corresponding ground-truth sequence where available.

Supplementary Movies - FVDM

Supplementary Movie S25. File: Supplementary_Movie_S25_FVDM_benign_case1.mp4. Method: FVDM. Class: Benign. Benign case 1 (case ID: 263b86b85a58f270). Format: MP4. This movie shows a breast ultrasound video sequence generated by the indicated method for qualitative comparison with the corresponding ground-truth sequence where available.

Supplementary Movie S26. File: Supplementary_Movie_S26_FVDM_benign_case2.mp4. Method: FVDM. Class: Benign. Benign case 2 (case ID: 2cda21c3aab26332). Format: MP4. This movie shows a breast ultrasound video sequence generated by the indicated method for qualitative comparison with the corresponding ground-truth sequence where available.

Supplementary Movie S27. File: Supplementary_Movie_S27_FVDM_benign_case3.mp4. Method: FVDM. Class: Benign. Benign case 3 (case ID: 32a6532065f51cec). Format: MP4. This movie shows a breast ultrasound video sequence generated by the indicated method for qualitative comparison with the corresponding ground-truth sequence where available.

Supplementary Movie S28. File: Supplementary_Movie_S28_FVDM_benign_case4.mp4. Method: FVDM. Class: Benign. Benign case 4 (case ID: 391ae00452fe2e99). Format: MP4. This movie shows a breast ultrasound video sequence generated by the indicated method for qualitative comparison with the corresponding ground-truth sequence where available.

Supplementary Movie S29. File: Supplementary_Movie_S29_FVDM_malignant_case1.mp4. Method: FVDM. Class: Malignant. Malignant case 1 (case ID: 1dc9ca2f1748c2ec). Format: MP4. This movie shows a breast ultrasound video sequence generated by the indicated method for qualitative comparison with the corresponding ground-truth sequence where available.

Supplementary Movie S30. File: Supplementary_Movie_S30_FVDM_malignant_case2.mp4. Method: FVDM. Class: Malignant. Malignant case 2 (case ID: 2a2716116cf2a1da). Format: MP4. This movie shows a breast ultrasound video sequence generated by the indicated method for qualitative comparison with the corresponding ground-truth sequence where available.

Supplementary Movie S31. File: Supplementary_Movie_S31_FVDM_malignant_case3.mp4.

Method: FVDM. Class: Malignant. Malignant case 3 (case ID: 2aef523b046e4671). Format: MP4. This movie shows a breast ultrasound video sequence generated by the indicated method for qualitative comparison with the corresponding ground-truth sequence where available.

Supplementary Movie S32. File: Supplementary_Movie_S32_FVDM_malignant_case4.mp4. Method: FVDM. Class: Malignant. Malignant case 4 (case ID: 2c12ff6464cfb1ff). Format: MP4. This movie shows a breast ultrasound video sequence generated by the indicated method for qualitative comparison with the corresponding ground-truth sequence where available.

Supplementary Movies - I2VGen

Supplementary Movie S33. File: Supplementary_Movie_S33_I2VGen_benign_case1.mp4. Method: I2VGen. Class: Benign. Benign case 1 (case ID: 263b86b85a58f270). Format: MP4. This movie shows a breast ultrasound video sequence generated by the indicated method for qualitative comparison with the corresponding ground-truth sequence where available.

Supplementary Movie S34. File: Supplementary_Movie_S34_I2VGen_benign_case2.mp4. Method: I2VGen. Class: Benign. Benign case 2 (case ID: 2cda21c3aab26332). Format: MP4. This movie shows a breast ultrasound video sequence generated by the indicated method for qualitative comparison with the corresponding ground-truth sequence where available.

Supplementary Movie S35. File: Supplementary_Movie_S35_I2VGen_benign_case3.mp4. Method: I2VGen. Class: Benign. Benign case 3 (case ID: 32a6532065f51cec). Format: MP4. This movie shows a breast ultrasound video sequence generated by the indicated method for qualitative comparison with the corresponding ground-truth sequence where available.

Supplementary Movie S36. File: Supplementary_Movie_S36_I2VGen_benign_case4.mp4. Method: I2VGen. Class: Benign. Benign case 4 (case ID: 391ae00452fe2e99). Format: MP4. This movie shows a breast ultrasound video sequence generated by the indicated method for qualitative comparison with the corresponding ground-truth sequence where available.

Supplementary Movie S37. File: Supplementary_Movie_S37_I2VGen_malignant_case1.mp4. Method: I2VGen. Class: Malignant. Malignant case 1 (case ID: 1dc9ca2f1748c2ec). Format: MP4. This movie shows a breast ultrasound video sequence generated by the indicated method for qualitative comparison with the corresponding ground-truth sequence where available.

Supplementary Movie S38. File: Supplementary_Movie_S38_I2VGen_malignant_case2.mp4. Method: I2VGen. Class: Malignant. Malignant case 2 (case ID: 2a2716116cf2a1da). Format: MP4. This movie shows a breast ultrasound video sequence generated by the indicated method for qualitative comparison with the corresponding ground-truth sequence where available.

Supplementary Movie S39. File: Supplementary_Movie_S39_I2VGen_malignant_case3.mp4. Method: I2VGen. Class: Malignant. Malignant case 3 (case ID: 2aef523b046e4671). Format: MP4.

This movie shows a breast ultrasound video sequence generated by the indicated method for qualitative comparison with the corresponding ground-truth sequence where available.

Supplementary Movie S40. File: Supplementary_Movie_S40_I2VGen_malignant_case4.mp4. Method: I2VGen. Class: Malignant. Malignant case 4 (case ID: 2c12ff6464cfb1ff). Format: MP4. This movie shows a breast ultrasound video sequence generated by the indicated method for qualitative comparison with the corresponding ground-truth sequence where available.

Supplementary Movies - LTX

Supplementary Movie S41. File: Supplementary_Movie_S41_LTX_benign_case1.mp4. Method: LTX. Class: Benign. Benign case 1 (case ID: 263b86b85a58f270). Format: MP4. This movie shows a breast ultrasound video sequence generated by the indicated method for qualitative comparison with the corresponding ground-truth sequence where available.

Supplementary Movie S42. File: Supplementary_Movie_S42_LTX_benign_case2.mp4. Method: LTX. Class: Benign. Benign case 2 (case ID: 2cda21c3aab26332). Format: MP4. This movie shows a breast ultrasound video sequence generated by the indicated method for qualitative comparison with the corresponding ground-truth sequence where available.

Supplementary Movie S43. File: Supplementary_Movie_S43_LTX_benign_case3.mp4. Method: LTX. Class: Benign. Benign case 3 (case ID: 32a6532065f51cec). Format: MP4. This movie shows a breast ultrasound video sequence generated by the indicated method for qualitative comparison with the corresponding ground-truth sequence where available.

Supplementary Movie S44. File: Supplementary_Movie_S44_LTX_benign_case4.mp4. Method: LTX. Class: Benign. Benign case 4 (case ID: 391ae00452fe2e99). Format: MP4. This movie shows a breast ultrasound video sequence generated by the indicated method for qualitative comparison with the corresponding ground-truth sequence where available.

Supplementary Movie S45. File: Supplementary_Movie_S45_LTX_malignant_case1.mp4. Method: LTX. Class: Malignant. Malignant case 1 (case ID: 1dc9ca2f1748c2ec). Format: MP4. This movie shows a breast ultrasound video sequence generated by the indicated method for qualitative comparison with the corresponding ground-truth sequence where available.

Supplementary Movie S46. File: Supplementary_Movie_S46_LTX_malignant_case2.mp4. Method: LTX. Class: Malignant. Malignant case 2 (case ID: 2a2716116cf2a1da). Format: MP4. This movie shows a breast ultrasound video sequence generated by the indicated method for qualitative comparison with the corresponding ground-truth sequence where available.

Supplementary Movie S47. File: Supplementary_Movie_S47_LTX_malignant_case3.mp4. Method: LTX. Class: Malignant. Malignant case 3 (case ID: 2aef523b046e4671). Format: MP4. This movie shows a breast ultrasound video sequence generated by the indicated method for qualitative comparison

with the corresponding ground-truth sequence where available.

Supplementary Movie S48. File: Supplementary_Movie_S48_LTX_malignant_case4.mp4. Method: LTX. Class: Malignant. Malignant case 4 (case ID: 2c12ff6464cfb1ff). Format: MP4. This movie shows a breast ultrasound video sequence generated by the indicated method for qualitative comparison with the corresponding ground-truth sequence where available.

Supplementary Movies - MCVD

Supplementary Movie S49. File: Supplementary_Movie_S49_MCVD_benign_case1.mp4. Method: MCVD. Class: Benign. Benign case 1 (case ID: 263b86b85a58f270). Format: MP4. This movie shows a breast ultrasound video sequence generated by the indicated method for qualitative comparison with the corresponding ground-truth sequence where available.

Supplementary Movie S50. File: Supplementary_Movie_S50_MCVD_benign_case2.mp4. Method: MCVD. Class: Benign. Benign case 2 (case ID: 2cda21c3aab26332). Format: MP4. This movie shows a breast ultrasound video sequence generated by the indicated method for qualitative comparison with the corresponding ground-truth sequence where available.

Supplementary Movie S51. File: Supplementary_Movie_S51_MCVD_benign_case3.mp4. Method: MCVD. Class: Benign. Benign case 3 (case ID: 32a6532065f51cec). Format: MP4. This movie shows a breast ultrasound video sequence generated by the indicated method for qualitative comparison with the corresponding ground-truth sequence where available.

Supplementary Movie S52. File: Supplementary_Movie_S52_MCVD_benign_case4.mp4. Method: MCVD. Class: Benign. Benign case 4 (case ID: 391ae00452fe2e99). Format: MP4. This movie shows a breast ultrasound video sequence generated by the indicated method for qualitative comparison with the corresponding ground-truth sequence where available.

Supplementary Movie S53. File: Supplementary_Movie_S53_MCVD_malignant_case1.mp4. Method: MCVD. Class: Malignant. Malignant case 1 (case ID: 1dc9ca2f1748c2ec). Format: MP4. This movie shows a breast ultrasound video sequence generated by the indicated method for qualitative comparison with the corresponding ground-truth sequence where available.

Supplementary Movie S54. File: Supplementary_Movie_S54_MCVD_malignant_case2.mp4. Method: MCVD. Class: Malignant. Malignant case 2 (case ID: 2a2716116cf2a1da). Format: MP4. This movie shows a breast ultrasound video sequence generated by the indicated method for qualitative comparison with the corresponding ground-truth sequence where available.

Supplementary Movie S55. File: Supplementary_Movie_S55_MCVD_malignant_case3.mp4. Method: MCVD. Class: Malignant. Malignant case 3 (case ID: 2aef523b046e4671). Format: MP4. This movie shows a breast ultrasound video sequence generated by the indicated method for qualitative comparison with the corresponding ground-truth sequence where available.

Supplementary Movie S56. File: Supplementary_Movie_S56_MCVD_malignant_case4.mp4. Method: MCVD. Class: Malignant. Malignant case 4 (case ID: 2c12ff6464cfb1ff). Format: MP4. This movie shows a breast ultrasound video sequence generated by the indicated method for qualitative comparison with the corresponding ground-truth sequence where available.

Supplementary Movies - Stable Video Diffusion XT (SVD-XT)

Supplementary Movie S57. File: Supplementary_Movie_S57_SVD_XT_benign_case1.mp4. Method: Stable Video Diffusion XT (SVD-XT). Class: Benign. Benign case 1 (case ID: 263b86b85a58f270). Format: MP4. This movie shows a breast ultrasound video sequence generated by the indicated method for qualitative comparison with the corresponding ground-truth sequence where available.

Supplementary Movie S58. File: Supplementary_Movie_S58_SVD_XT_benign_case2.mp4. Method: Stable Video Diffusion XT (SVD-XT). Class: Benign. Benign case 2 (case ID: 2cda21c3aab26332). Format: MP4. This movie shows a breast ultrasound video sequence generated by the indicated method for qualitative comparison with the corresponding ground-truth sequence where available.

Supplementary Movie S59. File: Supplementary_Movie_S59_SVD_XT_benign_case3.mp4. Method: Stable Video Diffusion XT (SVD-XT). Class: Benign. Benign case 3 (case ID: 32a6532065f51cec). Format: MP4. This movie shows a breast ultrasound video sequence generated by the indicated method for qualitative comparison with the corresponding ground-truth sequence where available.

Supplementary Movie S60. File: Supplementary_Movie_S60_SVD_XT_benign_case4.mp4. Method: Stable Video Diffusion XT (SVD-XT). Class: Benign. Benign case 4 (case ID: 391ae00452fe2e99). Format: MP4. This movie shows a breast ultrasound video sequence generated by the indicated method for qualitative comparison with the corresponding ground-truth sequence where available.

Supplementary Movie S61. File: Supplementary_Movie_S61_SVD_XT_malignant_case1.mp4. Method: Stable Video Diffusion XT (SVD-XT). Class: Malignant. Malignant case 1 (case ID: 1dc9ca2f1748c2ec). Format: MP4. This movie shows a breast ultrasound video sequence generated by the indicated method for qualitative comparison with the corresponding ground-truth sequence where available.

Supplementary Movie S62. File: Supplementary_Movie_S62_SVD_XT_malignant_case2.mp4. Method: Stable Video Diffusion XT (SVD-XT). Class: Malignant. Malignant case 2 (case ID: 2a2716116cf2a1da). Format: MP4. This movie shows a breast ultrasound video sequence generated by the indicated method for qualitative comparison with the corresponding ground-truth sequence where available.

Supplementary Movie S63. File: Supplementary_Movie_S63_SVD_XT_malignant_case3.mp4. Method: Stable Video Diffusion XT (SVD-XT). Class: Malignant. Malignant case 3 (case ID: 2aef523b046e4671). Format: MP4. This movie shows a breast ultrasound video sequence generated by

the indicated method for qualitative comparison with the corresponding ground-truth sequence where available.

Supplementary Movie S64. File: Supplementary_Movie_S64_SVD_XT_malignant_case4.mp4. Method: Stable Video Diffusion XT (SVD-XT). Class: Malignant. Malignant case 4 (case ID: 2c12ff6464cfb1ff). Format: MP4. This movie shows a breast ultrasound video sequence generated by the indicated method for qualitative comparison with the corresponding ground-truth sequence where available.

Supplementary Movies - Latte

Supplementary Movie S65. File: Supplementary_Movie_S65_Latte_benign_case1.mp4. Method: Latte. Class: Benign. Benign sample 1. Format: MP4. This movie shows a breast ultrasound video sequence generated by the indicated method for qualitative comparison with the corresponding ground-truth sequence where available.

Supplementary Movie S66. File: Supplementary_Movie_S66_Latte_benign_case2.mp4. Method: Latte. Class: Benign. Benign sample 2. Format: MP4. This movie shows a breast ultrasound video sequence generated by the indicated method for qualitative comparison with the corresponding ground-truth sequence where available.

Supplementary Movie S67. File: Supplementary_Movie_S67_Latte_benign_case3.mp4. Method: Latte. Class: Benign. Benign sample 3. Format: MP4. This movie shows a breast ultrasound video sequence generated by the indicated method for qualitative comparison with the corresponding ground-truth sequence where available.

Supplementary Movie S68. File: Supplementary_Movie_S68_Latte_benign_case4.mp4. Method: Latte. Class: Benign. Benign sample 4. Format: MP4. This movie shows a breast ultrasound video sequence generated by the indicated method for qualitative comparison with the corresponding ground-truth sequence where available.

Supplementary Movie S69. File: Supplementary_Movie_S69_Latte_malignant_case1.mp4. Method: Latte. Class: Malignant. Malignant sample 1. Format: MP4. This movie shows a breast ultrasound video sequence generated by the indicated method for qualitative comparison with the corresponding ground-truth sequence where available.

Supplementary Movie S70. File: Supplementary_Movie_S70_Latte_malignant_case2.mp4. Method: Latte. Class: Malignant. Malignant sample 2. Format: MP4. This movie shows a breast ultrasound video sequence generated by the indicated method for qualitative comparison with the corresponding ground-truth sequence where available.

Supplementary Movie S71. File: Supplementary_Movie_S71_Latte_malignant_case3.mp4. Method: Latte. Class: Malignant. Malignant sample 3. Format: MP4. This movie shows a breast ultrasound

video sequence generated by the indicated method for qualitative comparison with the corresponding ground-truth sequence where available.

Supplementary Movie S72. File: Supplementary_Movie_S72_Latte_malignant_case4.mp4. Method: Latte. Class: Malignant. Malignant sample 4. Format: MP4. This movie shows a breast ultrasound video sequence generated by the indicated method for qualitative comparison with the corresponding ground-truth sequence where available.

6. File location

Clearly labelled Supplementary Movie files (MP4) are provided in the folder "Supplementary_Movies". The original method-wise folder structure (Ground Truth, ours, CogVideo, FVDM, I2VGen, Latte, LTX, MCVD, SVD_XT) is also retained for convenience. An index table is saved as Supplementary_Movies/_legend_index.csv.

7. Quality and format statement

All Supplementary Movie files are supplied in MP4 format. Filenames explicitly encode the Supplementary Movie ID, method name, diagnostic class (benign/malignant), and case number.
